# Diseased Gums

**Published:** 1847-10

**Authors:** H. R. Smith

**Affiliations:** Terre Haute


***Diseased Gums, involved with general irritation of the mucous membrane of the Alementary canal, by*** H. R. Smith, D. D. S.

Terre Haute, September 25, 1847.

Doct. Taylor,

*Dear Sir*—Agreeable to your request I will give you the history of a few cases, which I have met with in my practice this summer, which are to me of a singular and interesting character ; and such as I have never seen described in any work which I have perused. I was at first somewhat baffled in my attempts at treatment, and am still at a loss how to account for the cause of the disease.

There has about twenty cases fallen into my hands, some of which had been treated by physicians and abandoned. In some cases it had an epidemic form, affecting every member in a family; at other times only one or two. I will give two or three cases which will embrace the general character of the whole, also my course of treatment.

Soon after my return from Cincinnati, in March last, I was called to see a lady, aged 35, of a scrofulous diathisis, but health generally good.

She told me that in January her gums commenced being irritable, and her teeth sensitive. The gums soon had the appearance of being under the influence of mercury, and she supposed that it was the effect of having taken mercury about one year ago. The gums gradually became swollen and spongy, and bled on the slightest touch.

Canker (for so I shall call it for want of a better name,) commenced about the fauces, and spread entirely over the mucous membrane of the mouth and tongue. At times the canker appeared in small white pimples, at others in elevated patches about the size of a half dime, with white margins. Again it would be one continued surface of white canker.

When I first saw her the canker had extended to the stomach, and whenever she received any food or fluids a severe burning sensation was produced in the stomach, which would last from one to two hours, causing considerable febrile excitement. The mucous membrane of the mouth seemed to be entirely destroyed ; a fœtid muco-purulent discharge from around the necks of all the teeth ; the teeth protruded from the alveola, very loose, and turning black ; the sublingual and submaxillary glands very much swollen, and painful on pressure, retching at the stomach and occasional vomiting, with a constant spitting of a thick, viscid matter or saliva ; at times a slight diarrhœa, and in the evening considerable vascular excitement.

Under these circumstances I commenced my treatment by removing the tartar which had collected in great quantities; extracting all badly diseased teeth and roots, scarrifying the gums freely; using warm water to aid the bleeding; rubbing the gums with a wash of tinc. myrrh, catechu and manna. For a week or more this treatment appeared to answer a good purpose, but the disease then put on even worse appearances than before. I then, in turn, tried various remedies, which have been recommended for similar cures, but with little benefit.

At last I concluded that it was more owing to constitutional than local derangement. I then tried the tonic treatment, recommended by Harris, for diseases of this nature, but without success. I at once changed my treatment entirely, and adopted the anti-philogistic course, restricting my patient to a low diet of vegetables, stale bread, milk and rice, cold or tepid mucilaginous drinks prohibiting tea and coffee, and food containing much salt or grease. Directed a gentle laxative every night; a gargle for the throat of Inf. gold thread, blackberry root and honey; a wash for the gums of tincture of myrrh, catechu, rhatany and honey, with free scarrifications and frictions of the gums every two or three days. This treatment was successful, and in about three months she was quite well, excepting her teeth still remaining very loose, and without a prospect of ever becoming firm.

The second case which I shall mention was the daughter of the lady above mentioned, aged ten years. The canker commenced opposite the first inferior molar of the right side, and in twenty-four hours, when I first saw her, the whole of the gum and membrane of the cheek, on the right side, was deeply involved. The submaxillary, sublingual and parotid glands much swollen and very painful.

Treatment, a brisk cathartic at first, and followed by the same treatment as the other, with a similar result, except considerable sloughing of the gum and internal muscles of the cheek.

Case third, A gentleman, aged thirty-eight, of good constitution, and health generally good. The disease commenced while he was at New Orleans on business, in January, similar to the first case, by swelling of the gums and canker about the fauces, came to my hands in April. Treatment, low diet, laxatives, mild tonics, and scarrification, friction and astringent washes, and, in this case, Nitrum Argentum to the gums. Result good, except considerable sloughing of the gums.

I could mention other cases, but I have already exceeded the limits which I intended, and as they are of a similar character it is unnecessary.

In some cases I found tonics beneficial, but for the most part I found a strict anti-phlogistic course the best. In nearly every case I had to extract more or less sound teeth, as they became so loose that they were of no use.

In one case I extracted four superior and six inferior front teeth, and all comparatively sound.

I would be happy to hear any suggestion that you or any other one might make in relation to the disease, or best course of treatment to pursue. Respectfully,

H. R. SMITH.

The communication from Dr. Smith, of Terre Haute, is of more than ordinary interest. The cases which he reports very strikingly illustrate the fact, that mere mechanical knowledge, however important to the Dentist, yet could be of little avail in treating those cases, where the soft parts, and indeed the whole system is so much affected by disease. We imagine that the epidemic form, which the disease in question assumed, was much the same as that which shows itself during the prevalence of Dysentery or bowel complaints, like causes operating on the same temperaments, will generally produce the same results, unless, indeed, constitutional vigor in one case may alter the type of disease, or entirely prevent its development.

The first case alluded to, appears to have been aggravated much by the presence of foreign matter about the teeth, and marks conclusively the effect which Dental irritation exerts on disease in general. It is probable that the younger member of the same family escaped the same prolongation of disease, because her teeth were less decayed, and not so much coated with tartar. The attack certainly was equally if not more severe. When we come to compare cases, however, it is necessary to know more of the particulars of each case. We take it for granted that persons so young as ten years, have not so much disease of the teeth, and accumulation of foeign matter around them.

The extensive irritation, indeed we might say inflammation, of the mucous coat of the alimentary canal, indicates the necessity of a mild antiphilogestic treatment, and we see this treatment conjoined to the local treatment of the mouth, that which most certainly relieved the patient, both were apparently essential to the cure, at all events the preservation of the teeth depended upon the treatment of the mouth.

It is very easy to account for the connection of diseased action in the gums. The glands which were involved in the disease, and the stomach, and also the consequent diarrhœa, nothing more is necessary than to take a correct anatomical view of the connection of those parts. We hope, by the issue of our next number, to get some further partrculars in relation to this disease from Dr. Smith—whether the teeth have since become carious in consequence of the disease—indicating an acid or irritated condition of the secretions of the mouth—whether the teeth of the children were much coated with tartar, and whether there exists a chronic form of diarrhœa in those cases where the teeth continue loose, and if so, see when, as this gives way to a proper treatment, with local tonics daily to the gums, keeping the teeth entirely free from extraneous matter, that they do not become more firm in their sockets. We should be glad to see such cases more frequently reported in Dental practice, and hope the Dr. will not only give us the promised case of malformation, but much else which his observation will force upon him.—Cin. Ed.

